# *Streptococcus suis* meningitis: An emerging zoonotic disease in Brazil

**DOI:** 10.1590/0037-8682-0610-2023

**Published:** 2024-04-05

**Authors:** Geisa Santos Souza Ramos, Renata Ferreira da Silva Rego, Maria Fernanda Feltrin de Oliveira, Verônica de França Diniz Rocha, Ernesto Pereira de Oliveira, Joice Neves Reis

**Affiliations:** 1 Universidade Federal da Bahia, Faculdade de Farmácia, Salvador, BA, Brasil.; 2 Instituto Couto Maia, Secretaria de Saúde do Estado da Bahia, Salvador, BA, Brasil.; 3 Escola Bahiana de Medicina e Saúde Pública, Fundação Bahiana para o Desenvolvimento das Ciências, Salvador, BA, Brasil.

**Keywords:** Streptococcus suis, Meningitis, Bacterial meningitis

## Abstract

*Streptococcus suis* has been widely reported as a pathogen in animals, especially pigs. In terms of human health implications, it has been characterized as a zoonosis associated with the consumption of pork products and occupational exposure, particularly in Southeast Asian countries. Here, we present a rare case of human *S. suis* infection in Brazil, diagnosed in an older adult swine farmer, a small rural producer residing in the semi-arid region of Bahia, Brazil.

## INTRODUCTION

*Streptococcus suis* is widely considered a pathogen affecting animals, especially pigs. It is a well-established cause of diseases, including meningitis, most frequently among suckling piglets, leading to a mortality rate of up to 20% if not treated. This bacterial species commonly colonizes animals' respiratory, digestive, and genitourinary tracts^1^. With respect to humans, it has been characterized as a zoonosis associated with handling colonized or infected animals, especially in Southeast Asian countries^2^.

In Brazil, studies conducted on farms in the states of São Paulo, Minas Gerais, and Paraná found that 58.8% of all pigs were infected with *S. suis*, mainly serotypes 2 and 9^3^ ^-^ ^5^. Recent cases of meningitis in humans have been reported to be associated with the consumption of pork products or occupational exposure^6^ ^,^ ^7^. 

Here, we report a case of *S. suis* meningitis that occurred in the semi-arid region of Bahia, Brazil. A literature review was performed using the Cochrane Library, LILACS, SciELO, MEDLINE, PubMed, and PubMed Central (PMC) databases and found only *S. suis* meningitis reported by Silva et al. and Ponte de Matos et al.^6^ ^,^ ^7^.

## CASE REPORT

A 68-year-old black male, married, and without comorbidities, was admitted to the emergency department of a private hospital in Feira de Santana, Bahia, Brazil, on September 27, 2019, with a five-day history of headache, fever, nausea, and vomiting. Neurological examination revealed nuchal rigidity and Brudzinski’s sign, while Kerning’s and Lasegue’s signs were absent. Fundoscopy revealed papilledema, whereas computed tomography of the skull revealed no acute changes. The white blood cell (WBC) count was 12,400 WBCs/μL, with 78% of the targeted polymorphonuclear (PMN) leukocytes. Upon establishing the meningitis hypothesis, a standard therapeutic regimen was initiated involving ceftriaxone (2 g IV 12/12h), ampicillin (2 g IV 4/4h), and dexamethasone (4 mg 6/6h). A cerebrospinal fluid (CSF) puncture was performed, confirming the diagnosis, with findings of 350 leukocytes/μL, a predominance of PMNs (89%), an elevated total protein count (119 mg/dL) in the CSF, and hypoglycorrhachia (32 mg/dL), along with evidence of gram-positive cocci grouped in pairs and short chains. Subsequently, ampicillin was discontinued. Blood cultures collected upon admission yielded negative results. 

Microbiological identification was established based on the growth of optochin-resistant alpha-hemolytic colonies on sheep blood agar, identified with 99.9% certainty by the VITEK 2 system as *Streptococcus suis* biotype 2. Matrix-assisted laser desorption ionization-time of flight confirmed the *S. suis* identification. 

After two days, on September 29, 2019, the patient was transferred to a reference state infectious diseases hospital in Salvador, Bahia, Brazil, for continued clinical treatment. During hospitalization, the patient experienced a bilateral decrease in hearing acuity, balance changes, and difficulty walking even with mobility aids. The patient exhibited grade 4 muscle strength in the lower limbs and grade 2 muscle strength in the upper limbs (per the classification reference).

The results of the additional tests are listed in Table 1. Serological tests for HIV, syphilis, and hepatitis B and C were negative. A second analysis of CSF revealed 150 leukocytes/μL with a predominance of mononuclear leukocytes, elevated protein levels (92 mg/dL), and decreased glucose levels (39 mg/dL).

Magnetic resonance imaging of the thoracic spine did not indicate the presence of myelitis. Following the diagnosis of meningitis caused by *S. suis*, ceftriaxone treatment alone was continued for 14 days.

During the entire hospitalization in the nursing facility, the patient consistently faced challenges such as reduced hearing acuity, difficulty walking, and diminished balance. Clinical and laboratory improvements were observed until discharge, as reflected by the sequential leukocyte counts shown in Table 1. Post-discharge deterioration in motor function was noted, further exacerbated by preexisting rheumatological and orthopedic conditions, including dorsal spondylosis and subscapular tendinopathy. The total hospitalization duration was 28 days.

**TABLE 1:** Clinical follow-up and summary of laboratory results.

	09/27/2019	09/29/2019	09/30/2019	10/03/2019	10/23/2019
**Blood parameters**					
Hemoglobin	-	14.9 g/dL	-	-	-
WBC count	12,400 WBCs/μL	14,170 WBC/μL	10,690 WBC/μL	9,790 WBC/μL	4,740 WBC/μL
Band PMNs	-	2%	0%	0%	-
Targeted PMNs	78%	81%	80%	60%	-
Platelets	-	288,000/μL	-	-	-
CRP	-	72.3 mg/L	-	-	-
Urea	-	68 mg/dL	-	-	-
Creatinine	-	1.10 mg/dL	-	-	-
Gamma-GT	-	48 UI/L	-	-	-
AST	-	21 UI/L	-	-	-
ALT	-	25 UI/L	-	-	-
Sodium	-	140 mEq/L	-	-	-
Potassium	-	4.5 mEq/L	-	-	-
HIV serology	-	Negative	-	-	-
Syphilis serology	-	Negative	-	-	-
HBV and HCV serologies	-	Negative	-	-	-
**CSF parameters**					
Leukocytes	350 leukocytes	150 leukocytes	-	-	-
PMNs	89%	Mononuclear cells predominant	-	-	-
CSF protein	119 mg/dL	92 mg/dL	-	-	-
CSF glucose	32 mg/dL	39 mg/dL	-	-	-
Lactate	8.4 mmol/L	-	-	-	-
Pandy test	-	Negative	-	-	-

**WBC:** White Blood Cells; **PMNs:** Polymorphonuclear Leukocytes; **CRP:** c-reactive protein; **GAMA-GT:** Gamma-glutamyl transferase; **AST:** Aspartate Transaminase; **ALT:** Alanine Transaminase; **HIV:** Human Immunodeficiency Virus; **HBV:** Hepatitis B virus; **HCV:** Hepatitis C virus; **CSF:** Cerebrospinal Fluid.

## DISCUSSION

Here, we report a case of *S. suis* meningitis occurring in the semi-arid region of Bahia, Brazil, where small rural families typically conduct pig farming without large-scale commercial production or modern technological practices. However, although the pig farm was family-operated, to improve its animal stock genetically, the farmer purchased piglets from breeders in southern Brazil, where there are many reports of animals colonized by *S. suis* ^3^. 

The primary risk factors for human infection include handling live or uncooked pigs and exposure to animal waste. Furthermore, direct contact with the mucous membranes of pets (such as cats and dogs), livestock (including horses and ruminants), and wild animals (such as deer and wild boars) may also constitute a significant source of infection^8^ ^,^ ^9^. Human *S. suis* infections are endemic in some countries, especially in Southeast Asia, where they are already recognized as occupational zoonosis^2^ ^,^ ^10^. In Brazil, cases of human infections have not been reported until recently. A verbal report of an occurrence in the interior of the state of Minas Gerais was preceded by a later report of a case associated with pork consumption involving an older person in Rio de Janeiro^6^. Subsequently, two additional cases of meningitis associated with the handling of pigs or pork products were described in the state of Ceará in northeastern Brazil^7^. Characteristics of meningitis cases attributed to *Streptococcus suis* identified in Brazil are outlined in Table 2.

**TABLE 2:** Characteristics of reported cases of Streptococcus suis meningitis in Brazil.

Characteristics	Case 1 (da Silva et al.^6^)	Case 2 (Ponte de Matos et al.^7^)	Case 3 (Ponte de Matos et al. ^7^)	Case 4 (this study)
Location	Rio de Janeiro	Ceará	Ceará	Bahia
Age (years)	82	60	68	68
Sex	Male	Male	Male	Male
Risk exposure	Consumption of porcine meat	Butcher	Farming (pig)	Farming (pig)
Comorbidities	Not reported	COPD/alcoholism	No	No
Clinical findings				
Fever	Yes	Yes	Yes	Yes
Neck stiffness	Not reported	Yes	Yes	Yes
Brudzinski	Not reported	Yes	No	No
Kernig	Not reported	No	No	No
Lasegue	Not reported	No	No	No
Treatment				
Antibiotic	Ceftriaxone + vancomycin + acyclovir (5 days)	Ceftriaxone (14 days)	Piperacillin/tazobactam (7 days)	Ceftriaxone + ampicillin (1 day)
	ceftriaxone alone (9 days)		Meropenem (14 days)	ceftriaxone alone (9 days)
			Polymyxin B (7 days)	
Corticosteroids	No	Yes	Yes	Yes
Sequalae	Not reported	Hearing loss	Not evident	Hearing loss
Outcome	Discharge	Discharge	Discharge	Discharge

COPD: chronic pulmonary obstructive disease.

We hypothesized that the geographic spread of *S. suis* infection might be attributed to the globalization of animal breeding practices, which involves importing breeding stocks and reproductive animals with superior genetic traits. In the present case, it was confirmed that the animals on the patient’s family farm originated from states in the southern region of Brazil, where cases of *S. suis* infection have previously been reported in animals^3^ ^,^ ^5^ ^,^ ^11^.

Although human infections most commonly manifest as acute meningitis, infections in other sites, including bloodstream and sepsis, have also been reported^10^. As in our patient, this infection generally responds well to standardized empirical treatment for streptococcal meningitis with ceftriaxone or penicillin, although sequelae, especially unilateral or bilateral hearing loss, have been reported. Proper use of corticosteroids may reduce the frequency and severity of sequelae^9^.

Since most laboratories in Brazil generally do not conduct phenotypic identification of this bacterium, healthcare professionals must remain vigilant concerning the differential microbiological diagnosis of meningitis caused by gram-positive cocci, especially in adults. When an isolated bacterium demonstrates resistance to optochin, a simple and low-cost test for the presumptive identification of *Streptococcus pneumoniae*, the primary target of differential diagnosis, should be performed. Although conventionally, *S. suis* demonstrates a highly favorable and stable sensitivity profile, routine antimicrobial susceptibility testing is essential for monitoring the potential emergence of antimicrobial resistance^2^ ^,^ ^12^.

We acknowledge certain limitations of our study, such as not sequencing the microorganism lineage and not conducting a microbiological investigation of the pigs at the farm. The lack of this information precludes the establishment of a molecular epidemiological relationship between the potential isolates from the pigs and the patient.

## CONCLUSION

The occurrence of human meningitis caused by *S. suis* should be considered a significant factor in the differential diagnosis of bacterial meningitis, especially in rural epidemiological contexts involving contact with pigs. As there are no vaccines for *S. suis*, prophylactic efforts to contain human infections typically rely on sanitation measures in pig-breeding facilities and the use of personal protective equipment by farmers and butchers handling pigs or pork products. For the general population, ensuring proper animal meat and viscera processing, including thorough cooking, is essential for prevention^2^. Adhering to the One Health approach, conducting microbiological and molecular investigations in animals associated with human cases is crucial to facilitate a comprehensive understanding of disease spread and control.

## ETHICS STATEMENT

The study protocol was approved by the Institutional Review Board of the Federal School of Pharmacy at the Federal University of Bahia (CAAE number 48107421.3.0000.8035) and granted final approval under number 4.879.603. The patient provided written informed consent.
